# Awareness of Temporal Lag is Necessary for Motor–Visual Temporal Recalibration

**DOI:** 10.3389/fnint.2015.00064

**Published:** 2016-01-05

**Authors:** Masaki Tsujita, Makoto Ichikawa

**Affiliations:** ^1^Graduate School of Advanced Integration Science, Chiba UniversityChiba, Japan; ^2^Japan Society for the Promotion of ScienceTokyo, Japan; ^3^Faculty of Letters, Chiba UniversityChiba, Japan

**Keywords:** lag adaptation, point of subjective simultaneity, temporal order judgment

## Abstract

Consistent exposure to a temporal lag between observers' voluntary action and its visual feedback induced recalibration of temporal order perception between a motor action and a visual stimulus. It remains unclear what kinds of processing underlie this motor–visual temporal recalibration. This study examined the necessity of awareness of a temporal lag between a motor action and its visual feedback for motor–visual temporal recalibration. In Experiment 1, we allocated observers to either the multiple-step or single-step lag conditions. In the multiple-step lag condition, we first inserted a small temporal lag and subsequently increased it with progress of the adaptation period, to make observers unaware of the temporal lag during the adaptation period. In the single-step lag condition, we instructed observers about the temporal lag before adaptation, and inserted a substantial temporal lag from the beginning of the adaptation period to ensure that they were aware of the temporal lag. We found significant recalibration only in the single-step lag condition. In Experiment 2, we exposed all observers to a substantial temporal lag from the beginning of adaptation period with no instruction about insertion of the temporal lag. We asked observers at the end of the experiment whether they were aware of the temporal lag. We found significant recalibration for only observers who were aware of the lag. These results suggest that awareness of the temporal lag plays a crucial role in motor–visual temporal recalibration.

## Introduction

When using optical or electrical devices, we are exposed to constant spatiotemporal discrepancy among sensory signals derived from different modalities. Under such circumstances, our perceptual system must compensate for the constant discrepancy to act adequately. Adaptive change to reduce the spatiotemporal discrepancy among modalities is one means of compensating for the discrepancy in such situations. Researchers have identified and examined such spatiotemporal adaptation. In the spatial domain, for instance, prolonged exposure to a constant spatial disparity between auditory and visual stimuli induces a spatial shift in auditory perception (ventriloquism aftereffect; Canon, 1970). Similarly, repeated pointing to a visual target through a prism induces spatial remapping between motor signals and visual feedback (prism adaptation; e.g., Stratton, 1897; Kohler, 1964; Epstein, 1975).

In the temporal domain, several studies have demonstrated adaptive compensation for temporal discrepancy between multiple sensory signals. Fujisaki et al. (2004) and Vroomen et al. (2004) independently reported recalibration of temporal perception between audition and vision. In their experiments, they exposed observers to a constant temporal lag between auditory and visual stimuli for a few minutes. This exposure to a constant temporal lag induced recalibration of audio–visual temporal perception. Results show that the point of subjective simultaneity (PSS) was shifted in the direction of compensating for the temporal lag in a subsequent temporal order judgment (TOJ) or simultaneity judgment (SJ) test between auditory and visual stimuli. Later studies demonstrated various detailed characteristics of temporal recalibration across sensory modalities. For instance, Hanson et al. (2008) replicated temporal recalibration in the TOJ task using various pairings of multisensory stimuli: audio–visual, audio–tactile, and visual–tactile stimuli. Di Luca et al. (2009) reported that audio–visual temporal recalibration transferred to audio–tactile or visual–tactile temporal perception in the TOJ task.

Recalibration arose not only in temporal perception between multiple sensory signals, but also in temporal perception between a voluntary action and its sensory feedbacks. Cunningham et al. (2001) carried the first report of motor–sensory temporal recalibration. They demonstrated that observers' performance of an obstacle avoidance task with a delayed visual feedback was increased by repetition of a trial with the delayed visual feedback. Moreover, they found negative aftereffect: adaptation to the delayed visual feedback reduced observers' performance of the subsequent obstacle avoidance task with no delay. Stetson et al. (2006) subsequently demonstrated motor–sensory temporal recalibration using a psychophysical method. They confirmed that a few minutes of exposure to a constant delayed visual feedback that was presented with observers' keypresses induced a shift of the PSS in the TOJ task between a voluntary keypress and a visual stimulus. Motor–sensory temporal recalibration has similar characteristics to those described above for multisensory recalibration. For instance, Heron et al. (2009) replicated motor–sensory temporal recalibration in TOJ and SJ tasks using various pairings of motor–sensory coordination: motor–visual, motor–auditory, and motor–tactile coordination. Moreover, Heron et al. (2009) and Sugano et al. (2010) demonstrated that motor–sensory temporal recalibration transferred to the temporal relationship between motor and the other sensory modality to which no temporal delay was introduced. Nevertheless, it remains unclear what kinds of processing underlie motor–sensory temporal recalibration.

Several studies of prism adaptation have revealed that spatial recalibration depends upon automatic processing that is driven without awareness of motor–visual spatial discrepancy. An example is the study conducted by Michel et al. (2007), which examined how awareness of the discrepancy influences prism adaptation. In their experiment, they allocated observers to either the multiple-step condition or the single-step condition. In the multiple-step condition, they gradually increased lateral visual shifts from 2 to 10 arc deg in steps of 2 arc deg by prisms during an adaptation period. Consequently, observers were unaware of the optical shift. In the single-step condition, as a consequence of exposure to a 10 arc deg lateral optical shift throughout the adaptation period, observers became spontaneously aware of the optical shift in the first trial of the adaptation period. They found that the extent of aftereffects in the multiple-step condition was greater than that in the single-step condition, even though the total extent of exposure to the visual shift in the multiple-step condition was less than that in the single-step condition.

Visual perception studies have also examined whether unaware presentation of adaptation stimuli influences aftereffects of visual adaptation. For instance, Wade and Wenderoth (1978) reported that the tilt aftereffect might be induced even if adaptation stimuli are invisible by binocular rivalry. In contrast, Moradi et al. (2005) reported that the face identity-specific aftereffect is canceled by binocular suppression or inattentional blindness of the inducing face. Furthermore, Maruya et al. (2008) reported that the motion aftereffect is partially affected by continuous flash suppression of a motion adaptor. In their study, the motion aftereffect was obtained despite unawareness of a motion adaptor when a stationary grating was presented as a test pattern (static test). However, the motion aftereffect was suppressed by unawareness of a motion adaptor only when a flickering grating was presented as a test pattern (dynamic test) to an eye where the motion adaptor was not presented. They concluded from their results that only low-level neurons could adapt to a motion even under an invisible adaptation condition. These findings suggest that awareness plays an important role in perceptual adaptation that depends upon a high-level processing where attended complex features are processed selectively, rather than perceptual adaptation that depends upon a low-level processing where simple features are processed automatically in parallel.

More recently, several reports described different characteristics between motor–sensory and multisensory temporal recalibration. On the one hand, Heron et al. (2012) demonstrated that audio–visual temporal order perception was recalibrated only at the spatial position where they presented visual stimuli during the adaptation period. On the other hand, Tsujita and Ichikawa (2012) demonstrated that motor–visual temporal recalibration is obtained independently of adapted spatial position. Considering that the neural response of visual processing corresponds well to the stimulus to the specific retinal or spatial positions (e.g., Tootell et al., 1982; Duhamel et al., 1997), these two studies imply that visual processing dependent upon retinal or spatial map is responsible for audio–visual temporal recalibration, but that higher-level processing than the visual processing underlies motor–visual temporal recalibration.

This study was conducted to clarify the mechanism underlying motor–sensory temporal recalibration by examining whether motor–visual temporal recalibration requires awareness of a temporal lag between a motor action and its sensory feedback. We would find significant recalibration even without awareness of the temporal lag if motor–sensory temporal recalibration depends upon the low-level processing like prism adaptation. In contrast, if the motor–sensory temporal recalibration depends upon the high-level processing which is related to awareness, then we would find significant recalibration only with awareness of the temporal lag.

## Experiment 1

In the first experiment, we operated observers' awareness of a temporal lag between observers' keypresses and visual feedback according to the procedure used by Michel et al. (2007). We allocated observers either to the multiple-step lag condition or single-step lag condition. In the multiple-step lag condition, during adaptation, we first inserted a slight temporal lag and increased it with the progress of an experimental session. We expected that observers were unaware of the temporal lag with the gradual increment. In contrast, in the single-step lag condition, we exposed them to a substantial temporal lag from the beginning of the adaptation period, and instructed the observers about inserting the temporal lag before the session, to make sure that they were aware of the temporal lag.

### Methods

#### Observers

Fifteen observers took part in Experiment 1. All had normal or corrected-to-normal vision. All were naïve to the purpose of the study. Seven and eight observers were assigned, respectively, to the multiple-step and single-step lag conditions. The experiment was approved by the local ethical committee of the department of psychology in Chiba University. Informed consent was obtained from each observer.

#### Stimuli and apparatus

Observers sat at a desk in a dimly lit and soundproof booth (KAWAI FKS20-12). They fixed their head on a chin rest 57 cm distance from a 17-inch CRT display (SONY CPD-G200J) with a refresh rate of 100 Hz. Stimuli were presented by a personal computer (Mac Pro 5.1 OS X 10.8.2) with Matlab 7.14 (MathWorks), using Psychophysics Toolbox extensions (Brainard, 1997; Pelli, 1997; Kleiner et al., 2007). As a fixation cross, a white cross (0.75 × 0.75 arc deg, 107 cd/m2) was presented at the center of the display on a black background (4 cd/m2). As a feedback and test flash, a white square (1.5 × 1.5 arc deg, 107 cd/m2) was flashed for one frame 2.25 arc deg above the fixation cross. A keyboard (Dell SK-8175 keyboard) on the desk was used for their keypresses. There was an inevitable delay between a keypress and a visual feedback (*M* = 58.5 ms, *SD* = 9.0 ms with 1000 measurements) because of our computer system. For convenience, we described the value of a temporal lag between a keypress and a visual feedback without adding the inevitable delay in the following text. Black cardboard covered their hands to prevent them from seeing their hands during the experiment. White noise (73 dB SPL) was presented continuously via headphones (SONY MDR-1R) to mask keypress sounds.

#### Procedure

A keypress training session in which observers learned an adequate pace of keypresses preceded the experimental session. The keypress training session comprised a first half and a second half. During the first half, before each trial, the white square was flashed six times with 1.54 Hz as a model of pace of keypresses. In each trial, observers were required to press a key six times with the same pace as the model. To correctly locate their fingers without seeing their hands, they put their index fingers on bumps on F and J keys. To avoid interference with keypress by the bump, they used the middle finger of their right hand for the keypresses. A sentence (“Too fast!” or “Too slow!”) was presented on the display as an alert about the pace of their keypresses if any of five intervals between six keypresses fell out of the range of 500–800 ms. The second half was almost identical to the first half, except that the model keypress pace was not presented before each trial. Therefore, they were required to press the key while remembering the model that was presented in the first half. They were allowed to finish each half if they could conduct 10 trials continuously without the alert. Consequently, they conducted at least 20 trials during the keypress training session.

**Figure 1:**
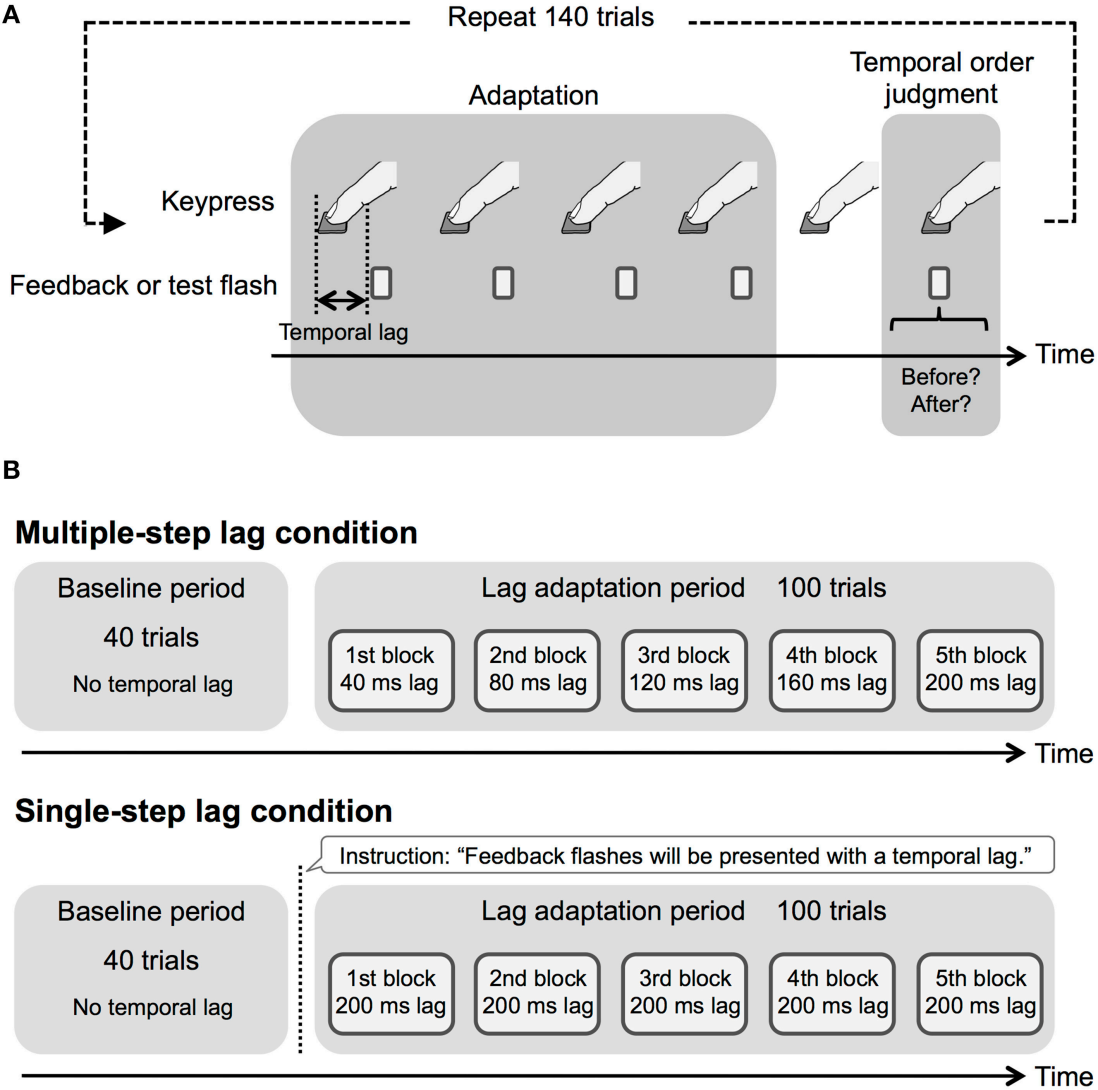
**Schematic illustration of procedure in Experiment 1**. **(A)** Experimental run and **(B)** experimental design in the experimental session.

In the experimental session, observers repeated a trial that included adaptation to a temporal lag and a TOJ test for the pair of a keypress and a flash (Figure 1A). Therefore, in each trial, they pressed the key six times with the pace learned during the keypress training session. From the first to fourth keypress, the feedback flash was presented with a fixed temporal lag from the keypresses. For the fifth keypress, no visual flash was presented to prevent the use of intervals between feedback flashes as a cue in the TOJ task. For the sixth keypress, the test flash was presented with one of the 10 test lag conditions (±15, 45, 75, 105, and 135 ms) from the predicted timing of the sixth keypress, which were derived from the averaged interval from the first to fifth keypresses. After six keypresses, they were required to respond whether the test flash was perceived before or after the sixth keypress (TOJ task). The alert sentence was presented on the display at the end of each trial as in the keypress training session if the pace of their keypresses was too fast or slow.

Observers conducted an easy dual task in each trial during the experimental session to confirm whether they had seriously engaged in the experiment. The fixation cross was extended to 1.5 × 1.5 arc deg for one frame once or twice with random timing between the first and fifth keypresses. After the TOJ response, observers were required to respond whether the fixation cross was extended once or twice.

Figure 1B shows the experimental design in the experimental session. Each observer conducted 140 trials in the experimental session. This number of trials was used to prevent observers from getting tired. We divided the experimental session into a baseline period (40 trials) and a lag adaptation period (100 trials). The baseline period preceded the lag adaptation period. During the baseline period, we presented the feedback flashes with no temporal lag. In the lag adaptation period, we divided 100 trials into five lag adaptation blocks (20 trials per block), and inserted the temporal lag according to the temporal lag conditions. Consequently, in the multiple-step lag condition, we respectively inserted 40, 80, 120, 160, and 200 ms temporal lag at the five blocks. In the single-step lag condition, we inserted 200 ms temporal lag throughout the five blocks. In addition, in the single-step lag condition only, we instructed the observers about inserting the temporal lag by displaying a description at the beginning of the lag adaptation period. Because the number of the trials at the baseline period and each lag adaptation block was insufficient to fit a psychometric function to each observer's data, we obtained psychometric functions by the use of all observers' data, as several previous studies on temporal order perception (e.g., Corveleyn et al., 2012).

Before the experimental session, we provided them with 10 practice trials so that they were familiar with the procedures. After the experimental session, we first asked the observers in the multiple-step lag condition a question whether anything changed during the experimental session. If the observers did not mention about the temporal lag between keypresses and feedback flashes, then we asked them whether they were aware of the temporal lag.

### Results

All the observers in the multiple-step lag condition reported that nothing changed during the experimental session in the first question after the experimental session, and reported that they were unaware of the temporal lag in the second question. Most observers in the two conditions showed high performance in the dual task during the experimental session (the mean of percentage correct of 93.8%, *SD* = 4.2%) except one observer in the single-step lag condition (percentage correct of 57.1%). Therefore, the data of this observer were excluded from further analysis. No significant difference was found in the percent correct for the dual task between two conditions [multiple-step lag, *M* = 95.5%, *SD* = 3.8%; single-step lag, *M* = 92.1%, *SD* = 4.1%; two-tailed *t*-test, *t*_(12)_ = 1.60, *n*.*s*.].

**Figure 2:**
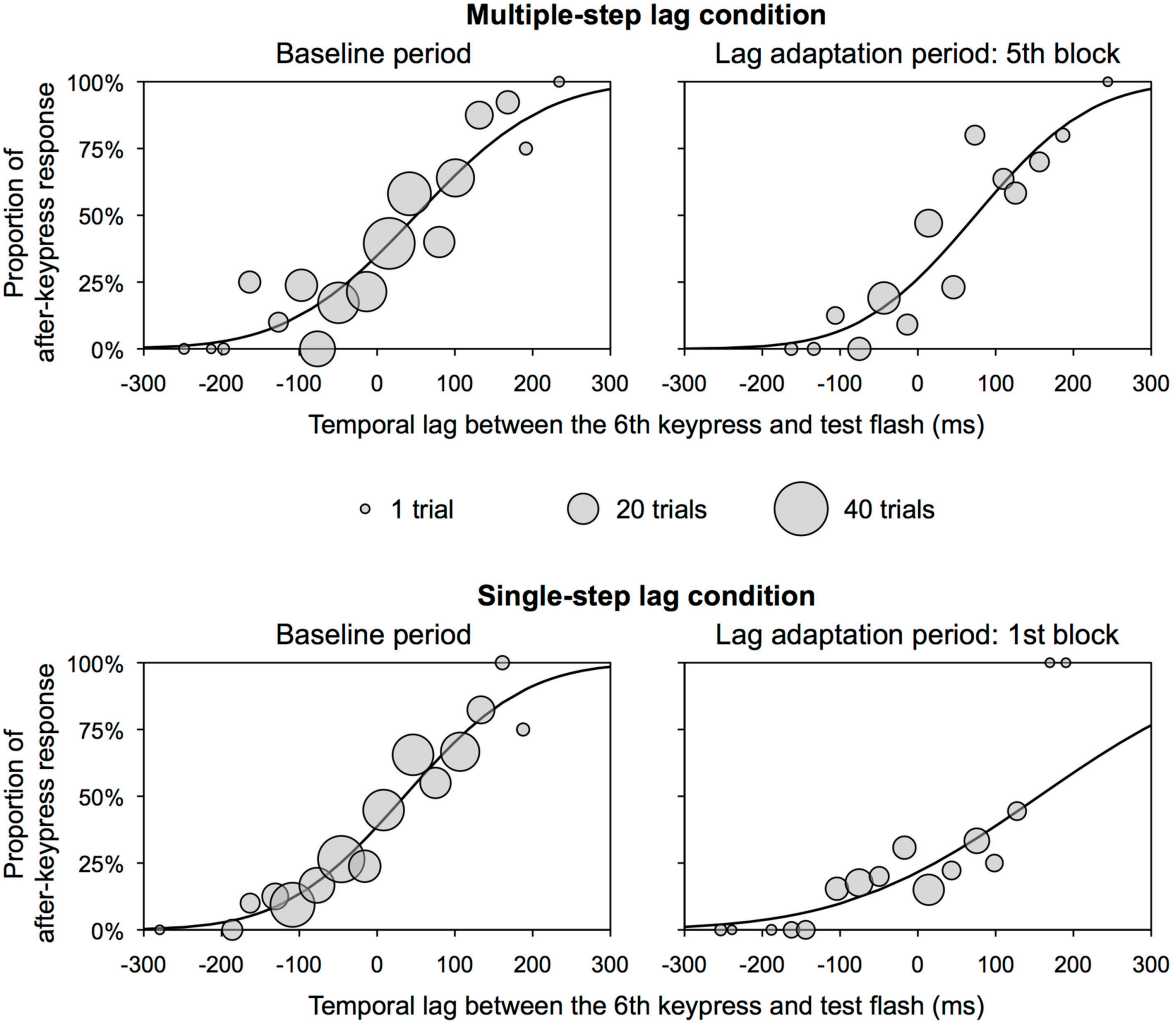
**Representative psychometric function for each condition in Experiment 1**. These functions were obtained by the use of all observers' data. Circles show the proportion of after-keypress response in each bin. Circle size shows the number of trials in each bin. A temporal lag between the sixth keypress and test flash on *x*-axis does not contain any inevitable delay due to a computer system.

We obtained a TOJ response and a temporal lag between a real sixth keypress and a test flash (positive values indicate that a test flash was presented after a sixth keypress) in each trial of the experimental session. The distribution of the temporal lag was scattered because of trial-to-trial variation between predicted and actual timing of a sixth keypress. We therefore binned the temporal lag into a 30 ms interval from -300 to 300 ms. Trials in which the temporal lag was beyond a range of -300 to 300 ms (0.5% of all trials) were excluded from further analysis. In each bin, we obtained a median of the temporal lags and a probability of a TOJ response that a test flash was perceived after a sixth keypress. We fitted a psychometric function to these values of the baseline period and each lag adaptation block using all observers' data, and obtained a 50% threshold as the PSS and the slope of the psychometric function by Probit analysis (Finney, 1971). The slope of the psychometric function is the reciprocal of the *SD* of the normal distribution. Small slope means low sensitivity in the TOJ. Figure 2 shows the representative psychometric functions at the baseline period and one of the lag adaptation blocks for each condition.

**Figure 3:**
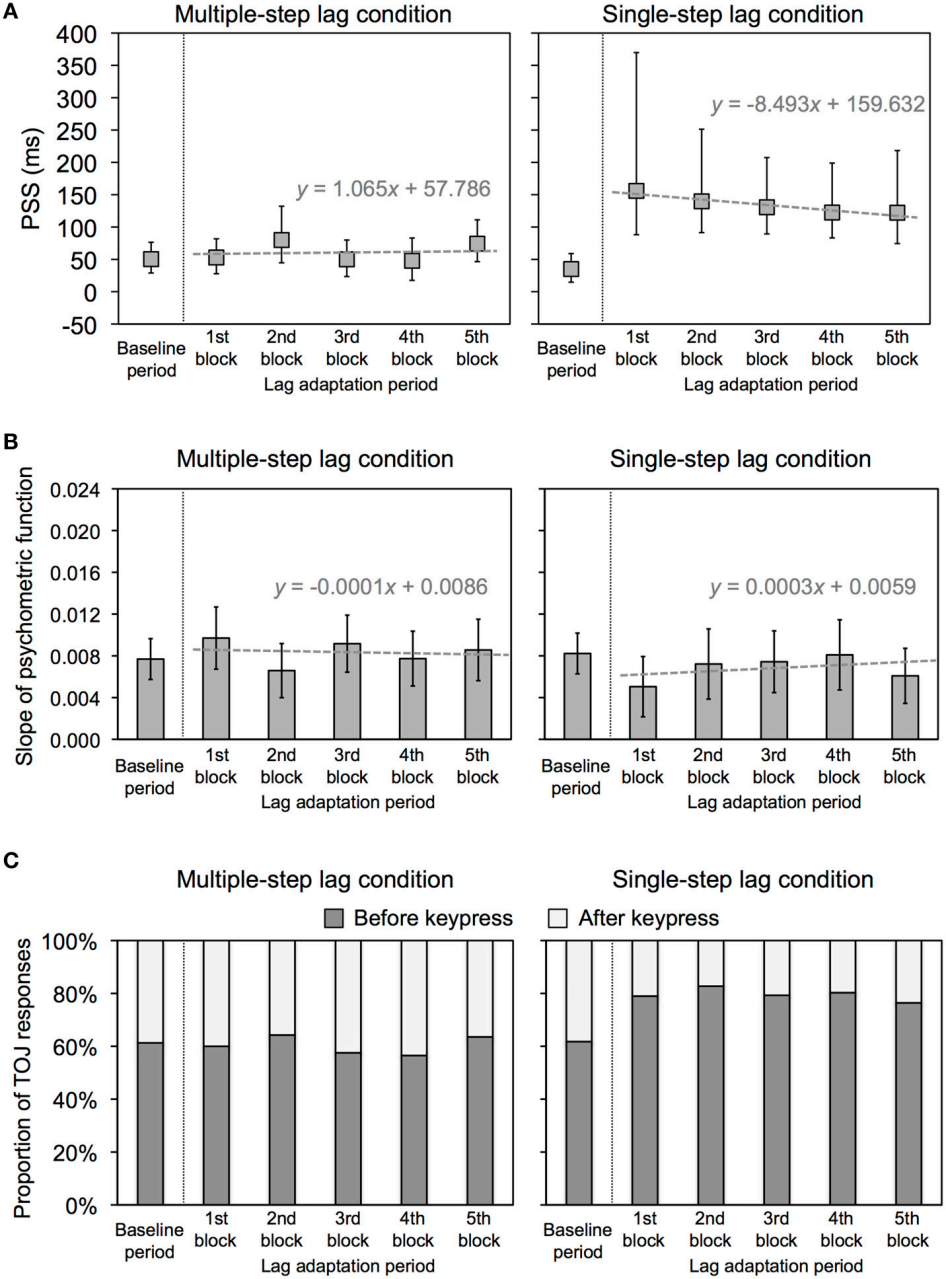
**Results of Experiment 1**. **(A)** PSS for multiple-step and single-step lag conditions in Experiment 1. Positive values in the PSS show that a test flash was presented after the sixth keypress. Error bars show a 95% confidence interval obtained from Probit analysis. Dotted gray lines show regression lines fitted to the PSSs at the lag adaptation blocks, with regression equations. **(B)** Slope of psychometric function for multiple-step and single-step lag conditions in Experiment 1. Error bars show a 95% confidence interval obtained from Probit analysis. Dotted gray lines show regression lines fitted to the PSSs at the lag adaptation blocks, with regression equations. **(C)** Proportion of TOJ responses for multiple-step and single-step lag conditions in Experiment 1. Dark and light gray bars respectively represent the response that a test flash was perceived before and after the sixth keypress.

Figure 3A shows the PSS and 95% confidence intervals, which were obtained in terms of Probit analysis for each condition. In the multiple-step lag condition, the PSSs at all lag adaptation blocks (the mean PSS was 61.0 ms) appeared to be similar to that at the baseline period (50.3 ms), even though temporal lags were inserted between keypresses and feedback flashes at those blocks. All of the 95% confidence intervals during the lag adaptation period overlapped with the 95% confidence interval at the baseline period. These results indicate that all the PSSs were constant through the experimental session in this condition (Figure 3A). In the single-step lag condition, the PSSs at all lag adaptation blocks (the mean PSS was 134.2 ms) were shifted to the temporal lag compared with that at the baseline period (35.0 ms). None of the 95% confidence intervals during the lag adaptation period overlapped with the 95% confidence interval at the baseline period. These results indicate that the PSSs shifted consistently during the lag adaptation period (Figure 3A).

We conducted linear regression analysis on the PSSs at all lag adaptation blocks to examine how the PSSs varied during the lag adaptation period (Figure 3A). In the multiple-step lag condition, the regression slope was not significantly different from zero [slope (*SE*) = 1.065 (5.416), *t*_(3)_ = 0.197, n.s.]. In the single-step lag condition, in contrast, the regression slope was significantly different from zero [slope (*SE*) = −8.493(1.622), *t*_(3)_ = −5.235, *p* = 0.014]. These results indicate that the PSSs shifted at the beginning of the lag adaptation period, but this shift gradually decreased with the progress of the lag adaptation period only in the single-step lag condition.

Figure 3B shows the slope of the psychometric function and 95% confidence intervals, which were obtained in terms of Probit analysis for each condition. All of the 95% confidence intervals during the lag adaptation period overlapped with 95% confidence interval at the baseline period in both two conditions. These results indicate that the sensitivity of the TOJ was constant through the experimental session in both two conditions. The regression slopes calculated by linear regression analysis on the slopes at all lag adaptation blocks were not significantly different from zero in both two conditions [multiple-step lag, slope (*SE*) = −0.0001 (0.0004), *t*_(3)_ = −0.254, n.s.; single-step lag, slope (*SE*) = 0.0003 (0.0004), *t*_(3)_ = 0.726, n.s.]. These results indicate that there was no consistent change for the sensitivity of the TOJ during the lag adaptation period in both two conditions.

Figure 3C shows the proportions of TOJ responses in each condition. Not only a shift of the PSS, but also change in the proportions of TOJ responses would reflect recalibration of temporal order perception. Comparing the proportion of TOJ responses at each lag adaptation block with that at the baseline period enable us a further statistical test as χ^2^ test, in addition to overlapping 95% confidence intervals of the PSSs. For the multiple-step lag condition, the χ^2^ test found no significant difference between the proportions of TOJ responses at the baseline period and every lag adaptation blocks (Table 1). This result of χ^2^ test indicates that the proportions of TOJ responses at the every lag adaptation blocks were unchanged from that at the baseline period. For the single-step lag condition, the χ^2^ test revealed that the proportions of TOJ responses at every lag adaptation block significantly differed from that at the baseline period (Table 1). This result of χ^2^ test indicates that the proportion of a before-keypress response at every lag adaptation blocks increased during the adaptation period compared with that at the baseline period.

**Table 1:** **Results of TOJ task in Experiment 1**.

	**Baseline period**	**Lag adaptation period**				
		**1st block**	**2nd block**	**3rd block**	**4th block**	**5th block**
**MULTIPLE-STEP LAG CONDITION**						
Before-keypress response	171	84	90	80	78	89
After-keypress response	108	56	50	59	60	51
$\chi_{\left(1\right)}^{2}$		0.07	0.36	0.54	0.87	0.21
**SINGLE-STEP LAG CONDITION**						
Before-keypress response	173	109	115	111	110	107
After-keypress response	107	29	24	29	27	33
$\chi_{\left(1\right)}^{2}$		12.46^**^	18.97^**^	13.05^**^	14.45^**^	9.01^**^

Double asterisks show values for which p < 0.01. For each condition, the first and second rows show the frequency of each response category in TOJ task. The third row shows $\chi_{\left(1\right)}^{2}$ from the relative frequency of each response category for the baseline period and each lag adaptation block.

### Discussion

Results in Experiment 1 show a clear difference in motor–visual temporal recalibration between the multiple-step and single-step lag conditions. Motor–visual temporal order perception was unchanged irrespective of inserting the temporal lag if the temporal lag was inserted gradually and the observers were unaware of the temporal lag. In particular, no significant recalibration was obtained at the fifth lag adaptation block even though a 200 ms temporal lag was inserted at the block. In contrast, if the temporal lag was substantially inserted from the beginning of the adaptation period, and if the observers were aware of the temporal lag by instruction about inserting the temporal lag, then the motor–visual temporal order perception was recalibrated in accordance with insertion of the temporal lag. These results suggest that awareness of the temporal lag plays an important role in motor–visual temporal recalibration.

However, the possibility exists that the recalibration was obtained only in the single-step lag condition because of other factors. For instance, the total extent of exposure to the temporal lag might be too small to achieve recalibration in the multiple-step lag condition. In fact, the trials with 200 ms temporal lag in the multiple-step lag condition (20 trials in total) were considerably fewer than those in the single-step lag condition (100 trials in total). One might expect that, if the same exposure to the temporal lag as in the single-step lag condition is provided, then recalibration could be obtained even without awareness of the temporal lag. In addition, instruction about the temporal lag might cause artifacts in the single-step lag condition: the unnatural instruction might force observers to bias attention to something or to infer the experimenters' expectation. Consequently, observers might have changed their judgments. In Experiment 2, we reexamined the influence of awareness of the temporal lag to motor–visual recalibration under a condition that the total extent of the exposure was equated and that no instruction about the temporal lag was provided.

## Experiment 2

In the second experiment, we exposed all observers to a substantial temporal lag from the beginning of the lag adaptation period, as in the single-step lag condition in Experiment 1, except that we did not instruct them about inserting the temporal lag before adaptation. Furthermore, we asked observers whether they were aware of the temporal lag after the experimental session, and classified them as unaware or aware of lag groups based upon their reports. Consequently, the total extent of exposure to the temporal lag was equal between these two groups, and no instruction about the temporal lag was provided for either group. Therefore, comparing between these two groups enabled us to examine clearly whether motor–visual temporal recalibration required awareness of the temporal lag.

### Methods

Eleven new observers participated in Experiment 2. All had normal or corrected-to-normal vision. All were naïve to the purpose of the study. The experiment was approved by the local ethical committee of the department of psychology in Chiba University. Informed consent was obtained from each observer. The stimuli, apparatus, and procedures were the same as those in the single-step lag condition in Experiment 1, except that no instruction of inserting temporal lags was provided before the lag adaptation period. In addition, after the experimental session, we asked the observers the same questions as those in the multiple-step lag condition in Experiment 1.

### Results and discussion

Two observers reported that feedback flashes were delayed halfway during the experimental session in the first question after the experimental session. Another two observers reported that they were aware of the temporal lag in the second question. These four observers' data were treated as the aware of lag group in further analysis. The other seven observers who were unaware of the temporal lag were treated as the unaware of lag group in further analysis. All the observers showed high performance in the dual task during the experimental session (the mean of percentage correct of 92.0%, *SD* = 6.9%). There was no significant difference in the percent correct for the dual task between two groups [unaware of lag group, *M* = 90.4%, *SD* = 8.3%; aware of lag group, *M* = 94.8%, *SD* = 2.3%; two-tailed *t*-test, *t*_(9)_ = 1.02, *n*.*s*.].

**Figure 4:**
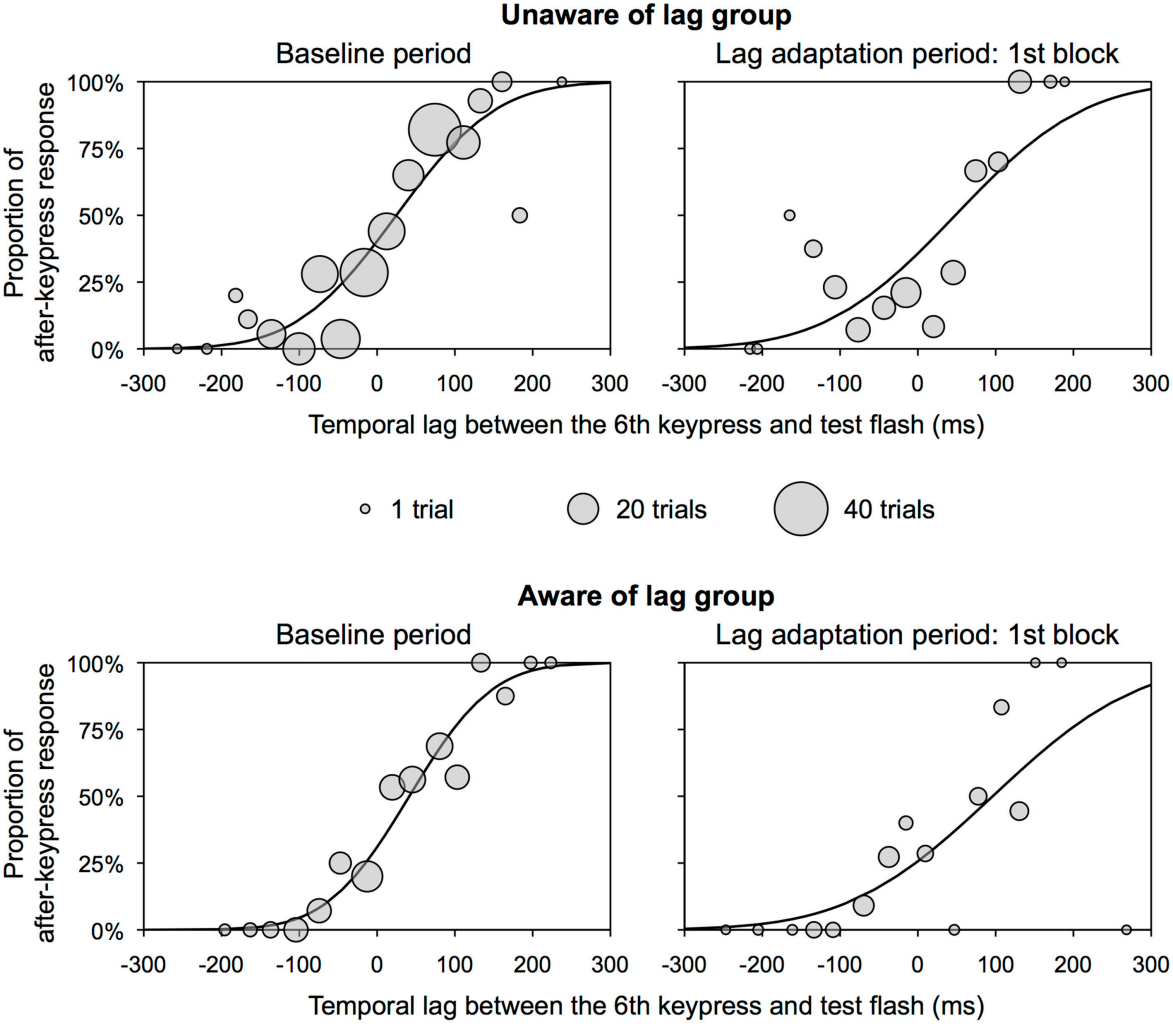
**Representative psychometric function for each group in Experiment 2**. These functions were obtained by the use of all observers' data. Circles show the proportion of after-keypress response in each bin. Circle size shows the number of trials in each bin. A temporal lag between the sixth keypress and test flash on *x*-axis does not contain any inevitable delay due to a computer system.

**Figure 5:**
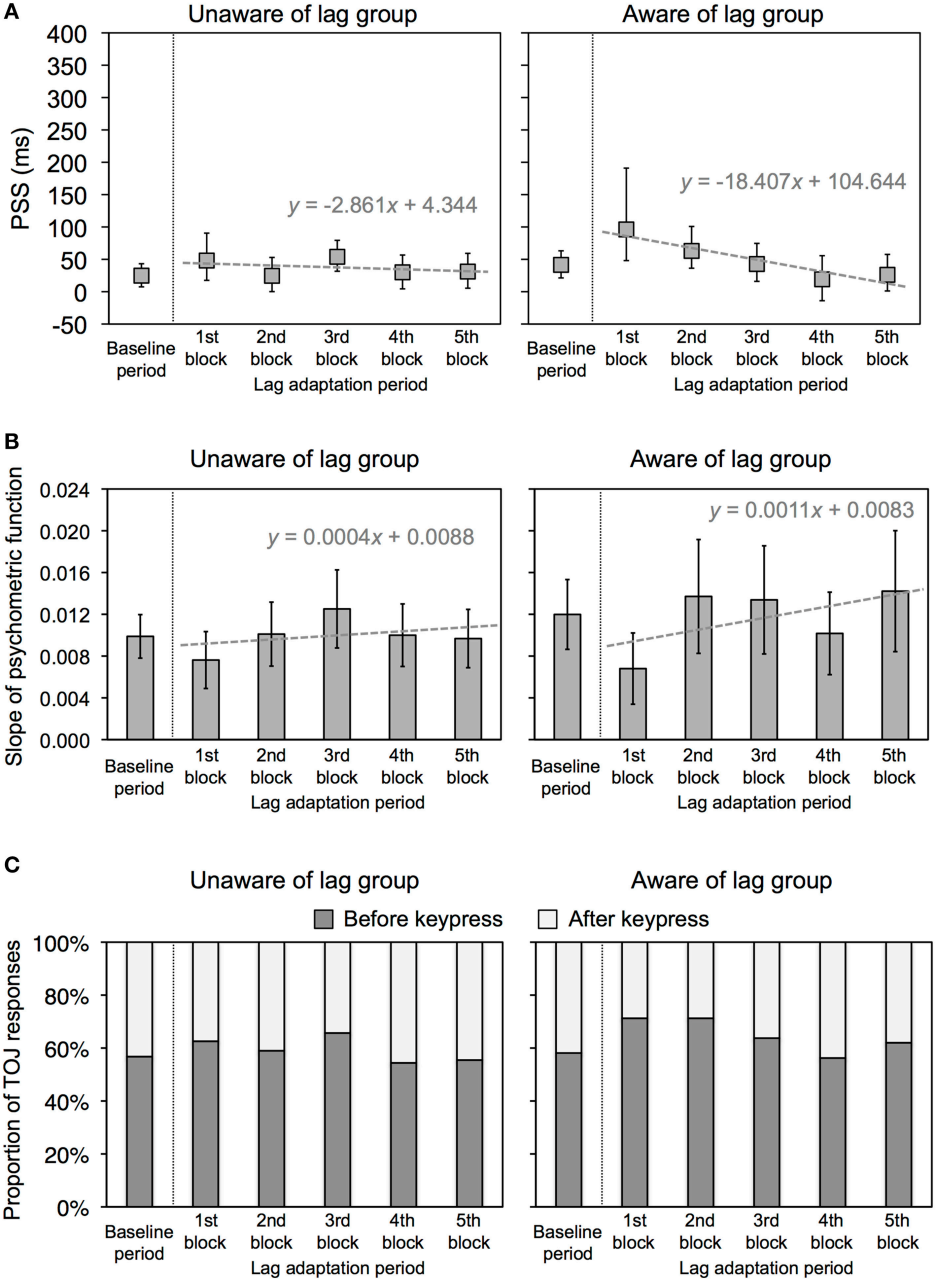
**Results of Experiment 2**. **(A)** PSS for unaware and aware of lag groups in Experiment 2. Positive values in the PSS show that a test flash was presented after the sixth keypress. Error bars show a 95% confidence interval obtained from Probit analysis. Dotted gray lines show a regression line fitted to the PSSs at the lag adaptation blocks, with a regression equation. **(B)** Slope of psychometric function for unaware and aware of lag groups in Experiment 2. Error bars show a 95% confidence interval obtained from Probit analysis. Dotted gray lines show a regression line fitted to the PSSs at the lag adaptation blocks, with a regression equation. **(C)** Proportion of TOJ responses for unaware and aware of lag groups in Experiment 2. Dark and light gray bars respectively represent responses that a test flash was perceived before and after the sixth keypress.

The PSS and slope were obtained using the same analysis as that in Experiment 1. Figure 4 shows the representative psychometric functions at the baseline period and one of the lag adaptation blocks for each group. Excluded trials in binning were 0.8% of all trials. Figure 5A shows the PSS for each group. In the unaware of lag group, the PSSs at all lag adaptation blocks (the mean PSS was 37.5 ms) were close to that at the baseline period (24.8 ms). In the aware of lag group, similarly, the PSSs at all lag adaptation blocks (the mean PSS was 49.4 ms) were close to that at the baseline period (41.4 ms). The 95% confidence intervals of the PSSs at every lag adaptation blocks overlapped with that at the baseline period in both two groups.

Figure 5B shows the slope of the psychometric function and 95% confidence intervals for each group. The 95% confidence intervals at every lag adaptation blocks overlapped with that at the baseline period in both two groups. These results indicate that the sensitivity of the TOJ was constant through the experimental session in both two groups. The regression slopes calculated by linear regression analysis on the slopes at all lag adaptation blocks were not significantly different from zero in both two groups [unaware of lag, slope (*SE*) = 0.0004 (0.0005), *t*_(3)_ = 0.679, n.s.; aware of lag, slope (*SE*) = 0.0011 (0.0009), *t*_(3)_ = 1.195, n.s.]. These results indicate that the sensitivity of the TOJ during the lag adaptation period did not change consistently in both two groups.

Figure 5A shows that the PSSs were high at the beginning of the lag adaptation period and decreased with the progress of the lag adaptation period in the aware of lag group. The decrement of the PSS was confirmed by the result that the regression slope calculated by linear regression analysis on the PSSs at all lag adaptation blocks was significantly different from zero [slope (*SE*) = −18.407(4.039), *t*_(3)_ = −4.557, *p* = 0.020]. The PSS shift at the beginning of the lag adaptation period was confirmed by the χ^2^ test that the proportions of TOJ responses at the first and second lag adaptation blocks significantly differed from that at the baseline period (Table 2, Figure 5C). In the unaware of lag group, in contrast, the regression slope of the PSSs at all lag adaptation blocks was not significantly different from zero [slope (*SE*) = −2.861 (4.344), *t*_(3)_ = −0.659, n.s.]. The χ^2^ test found no significant difference between the proportions of TOJ responses at the baseline period and every lag adaptation blocks (Table 2, Figure 5C).

**Table 2:** **Results of TOJ task in Experiment 2**.

	**Baseline period**	**Lag adaptation period**				
		**1st block**	**2nd block**	**3rd block**	**4th block**	**5th block**
**UNAWARE OF LAG GROUP**						
Before-keypress response	159	87	82	90	74	76
After-keypress response	121	52	57	47	62	61
$\chi_{\left(1\right)}^{2}$		1.29	0.19	3.03	0.21	0.06
**AWARE OF LAG GROUP**						
Before-keypress response	93	57	57	51	45	49
After-keypress response	67	23	23	29	35	30
$\chi_{\left(1\right)}^{2}$		3.92^*^	3.92^*^	0.70	0.08	0.33

Asterisks show values for which p < 0.05. For each condition, the first and second rows show the frequency of each response category in TOJ task. The third row shows $\chi_{\left(1\right)}^{2}$ from the relative frequency of each response category for the baseline period and each lag adaptation block.

It is evident from the results of the linear regression analysis on the PSSs and the χ^2^ test on the proportion of TOJ responses that awareness of the temporal lag affected motor–visual temporal recalibration even though the total extent of exposure to the temporal lag was equal between the two groups, and no instruction of inserting the temporal lag was provided for either group. Motor–visual temporal order perception was recalibrated at the beginning of the lag adaptation period and reverted to its state in the baseline period by degrees if observers were aware of the temporal lag. If not, the motor–visual temporal order perception remained unchanged.

## General discussion

Two experiments demonstrated that awareness of a temporal lag between the observer's motor action and its visual feedback plays an important role for motor–visual temporal recalibration. In Experiment 1, recalibration was obtained only if observers were aware of the temporal lag by substantially inserting the temporal lag and instructing observers about the temporal lag. In Experiment 2, recalibration was obtained only if observers were aware of the temporal lag, as in Experiment 1, even if the observers were exposed to the same motor–visual temporal discrepancy and were not instructed about the temporal lag. These results in Experiment 2 eliminate the possibility that absence of the total extent of exposure to the temporal lag or experimental artifacts by instructing about the temporal lag caused significant recalibration in the single-step lag condition in Experiment 1.

Someone may suspect that the obtained PSS shift for observers who were aware of the temporal lag in both two experiments was caused by a response bias that arose from knowledge of the temporal lag, which was introduced by instruction (Experiment 1), or by perceptual detection of the temporal lag (Experiment 2). Such a suspect would be based on the assumption that those observers reported more before-keypress response in the TOJ task regardless of their perception. In fact, several studies indicate that the TOJ task was susceptible to response biases (Schneider and Bavelier, 2003; Van Eijk et al., 2008). This account for the PSS shift in terms of response bias can be denied by two results in the present experiments. First, the results of the two experiments showed that the PSS shift during the lag adaptation period significantly decreased with progress of the period when observers were aware of the temporal lag. If the PSS shift were induced by a response bias derived from knowledge of the temporal lag, constant PSS shift should be obtained throughout the lag adaptation period. Second, no explicit difference between the slope of the psychometric function at the baseline period and the lag adaptation period was obtained in the two experiments when observers were aware of the temporal lag. If observers uniformly reported more before-keypress response regardless of a temporal lag between a sixth keypress and a test flash because of the response bias in terms of the knowledge of the temporal lag, the proportion of a before-keypress response in every bins should increase, and the slope during the lag adaptation period should be gentler than that at the baseline period.

The obtained recalibration in the aware of lag group in Experiment 2 was modest compared to that in the multiple-step lag condition in Experiment 1. Presumably, such difference was a consequence of uncertainty of awareness of the temporal lag for observers who were classified into the aware of lag group in Experiment 2. In Experiment 1, the observers who were assigned to the single-step lag condition were certainly aware of the temporal lag because of instruction they received about the temporal lag. In contrast, in Experiment 2, the observers in the aware of lag group were uncertain about the temporal lag because they were not instructed about it. In fact, some observers in the aware of lag group reported in the question after the experimental session that they did not know when the temporal lag was inserted or how long it lasted, although they were somehow aware of the temporal lag. The decrement of PSS shift during the lag adaptation period for observers who were aware of lag in both two experiments would be explained by certainty of awareness of the temporal lag. That is, explicit recalibration was primarily induced by awareness of the temporal lag at the beginning of the lag adaptation period. However, the recalibration subsequently attenuated with progress of the lag adaptation period because of uncertain awareness of the temporal lag by decrease of perceived temporal lag derived from the recalibration itself. One of the limitations in the present study is that we can only guess whether and how the observers were aware of the temporal lag based upon their report after the experimental session. It will be better for further study to examine the influence of awareness of the temporal lag on motor–visual temporal recalibration by the use of individual detection thresholds for the temporal lag.

We obtained temporal recalibration in two experiments with temporal fluctuation between a keypress and a visual feedback (*SD* = 9.0 ms) by the use of a usual keyboard. One may suspect that this fluctuation would affect adaptation to a temporal lag between a keypress and a visual feedback. However, Yamamoto and Kawabata (2011) demonstrated that adaptation to a fluctuated temporal lag between motor and auditory feedback [combining 66 and 133 ms (*SD* = 33 ms), or 33 and 166 ms (*SD* = 66 ms)] induced temporal recalibration to the same degree as that in adaptation to the consistent temporal lag [constantly 100 ms (*SD* = 0 ms)]. Their results indicate that, even with fluctuation of the temporal lag, mean of the temporal lag is responsible for the motor–auditory temporal recalibration. Although Yamamoto and Kawabata (2011) combined motor with auditory feedback, in line with their results, we may expect that the obtained temporal recalibration in the present two experiments with temporal fluctuation (*SD* = 9.0 ms) between motor and visual feedback is at the same level as the one obtained with more accurate keyboard. In addition, the slope of the psychometric curve, which is estimated as SD in TOJ task, might be gentle by the use of the inaccurate keyboard. Nevertheless, the relative relationship in the slopes between blocks or conditions would be not affected by the temporal precision of the keyboard because we used the same keyboard in all conditions and periods.

Altogether, the present results indicate that awareness of the temporal lag plays an important role in motor–visual temporal recalibration. These results enable us to discuss what kinds of processing contribute to motor–visual temporal recalibration. As described in the *Introduction* of this report, awareness of adaptation stimuli is necessary for many perceptual adaptations that depend upon high-level processing (Moradi et al., 2005; Maruya et al., 2008), and not those that depend upon low-level processing (Wade and Wenderoth, 1978; Maruya et al., 2008). Considering these matters, the present results imply that motor–visual temporal recalibration depends upon high-level processing in which awareness is involved in the registration of the temporal lag for adaptive processing.

Previous studies proposed mechanisms that underlie temporal recalibration. Among them, two models by Roach et al. (2011) and Cai et al. (2012) are apparently more plausible because both models are based upon neural architecture that underlies various perceptual mechanisms. On the one hand, Roach et al. (2011) emphasized low-level neural population codes that fire maximally at a particular temporal lag between visual and auditory stimuli, and advocated that audio–visual temporal recalibration results from the decrease of response gain of the neurons that are most sensitive to the adapted temporal lag. The possibility exists that the model also underlies motor–sensory temporal recalibration because neurons that were tuned to a particular temporal lag in a multisensory stimulus were also found in the somatosensory–visual and somatosensory–auditory domain in cats (Meredith et al., 1987). On the other hand, Cai et al. (2012) assumed that outputs of the low-level neural population codes feed into high-level rivaling neural populations (encoding “before” and “after” the motor action), and that the difference of activity between these populations determines the perceptual judgment. They advocated that consistent exposure to a delayed feedback decreases the input weights of the “after” module and increases that of the “before” module because of synaptic scaling of their modules, and that consistent exposure to a delayed feedback produces a perceptual decision bias to “before” judgments. Which of these models fits the present results?

The present results demonstrated that consistent exposure to the temporal lag induced recalibration only if observers were aware of the temporal lag. These results disagree with the model proposed by Roach et al. (2011) because the low-level neural population coding that they assumed is a system in which various temporal lags can be processed automatically in parallel. Consistent exposure to the temporal lag should induce the recalibration automatically even though observers were unaware of the temporal lag if the model by Roach et al. (2011) underlies motor–visual temporal recalibration. Rather, the present results agree with the model proposed by Cai et al. (2012) because high-level rivaling neural populations that they assumed constitute a system to achieve a perceptual judgment or decision: unawareness of the temporal lag involves inactivation of these neural populations. Therefore, if the model by Cai et al. (2012) underlies recalibration, then exposure to the temporal lag is expected to induce recalibration only with awareness of the temporal lag.

The registration of the temporal lag with awareness in high-level processing is reasonable for the appropriate usage of various sources of information related to voluntary actions. When we execute some sort of voluntary action, we acquire both pre-movement signals (premotor signals or copies of efferent motor signals) and various reafferent signals as sensory feedback (e.g., visual, auditory, somatosensory, and tactile signals from a part of body), and would perceive the onset time of the action from their signals (Libet et al., 1983; Haggard et al., 1999; Obhi, 2007; Obhi et al., 2009). Consequently, numerous temporal relationships exist among various signals related to the action. Therefore, it is necessary to select a proper temporal relationship from various temporal relationships by registration with awareness to realize appropriate recalibration.

The present study provides insight into different mechanisms of motor–sensory and multisensory temporal recalibration. Many of previous studies have demonstrated that voluntariness of motor actions influences various aspects of perception in terms of sensory consequences (e.g., Blakemore et al., 1998; Haggard et al., 2002). Furthermore, several studies on temporal recalibration, as described in the *Introduction* of this report, revealed that recalibration is induced independently of an adapted retinal position in the motor–visual domain (Tsujita and Ichikawa, 2012), but not in the audio–visual domain (Heron et al., 2012). This difference between motor–visual and audio–visual recalibration suggests that the responsible processing for the motor–sensory temporal recalibration is relatively higher than that for the multisensory temporal recalibration (Tsujita and Ichikawa, 2012). The present results support this notion. Multisensory temporal recalibration entails a small number of temporal relationships between multisensory signals from an external event caused by no voluntary action. Therefore, it is efficient that exposure to a temporal lag between multisensory signals automatically induced recalibration without registration with awareness because there is no need to select a temporal relationship that should be recalibrated.

A recent report described that audio–visual temporal recalibration was induced only with awareness (Gallagher et al., 2014). These results are opposed to the idea that multisensory temporal recalibration was induced automatically without registration with awareness. It is noteworthy that Gallagher et al. (2014) removed awareness of an audio–visual temporal lag by the use of dichoptic presentation of a visual stimulus. Consequently, observers in their study were not only unaware of a temporal lag between a visual and auditory stimulus; they were also unaware of a visual stimulus, which should be coupled with the auditory stimulus. Considering these points, the possibility exists that absence of recalibration in Gallagher et al. (2014) results from an absence of interaction between vision and audition by subliminal presentation of a visual stimulus, rather than by unawareness of a temporal lag between a visual and auditory stimulus. The influence of awareness of a temporal lag in multisensory temporal recalibration should be examined in a future study using the same experimental paradigm as that in this study.

Pesavento and Schlag (2006) examined motor–sensory temporal recalibration in adaptation to a progressively delayed visual feedback, which is similar to the multiple-step lag condition of Experiment 1 in the present study. Surprisingly, they found negative aftereffect of the adaptation even though observers were unaware of a delay between keypresses and visual feedbacks. A contradiction of results between the present study and Pesavento and Schlag's study might be carried by a difference of task during an adaptation period. In Pesavento and Schlag (2006), observers were required to press a key with a delayed visual feedback in synchrony with isochronous sequence of visual flashes as targets during an adaptation period. To conduct this task, observers would gather an error between the feedback and target in each keypress, and would use the error for adequate temporal control of a keypress in the next keypress. Consequently, observers would recalibrate motor–visual temporal perception based upon the error without awareness of the delay between keypresses and visual feedbacks. In the present experiment, observers were required to press a key with constant pace during the lag adaptation period without a target with which observers should synchronize visual feedback. Consequently, observers were unable to use an error between the visual feedback and target for adequate control of the next keypress, and would recalibrate motor–visual temporal perception based only upon awareness of a temporal lag between a motor action and its visual feedback.

This account in terms of an error between a feedback and target can apply to an opposite characteristic of motor–visual temporal recalibration to that of prism adaptation: unawareness of a temporal discrepancy between a motor action and visual feedback caused a lack of temporal recalibration in this study, whereas unawareness of a spatial discrepancy between motor action and visual feedback increased aftereffects of prism adaptation (Michel et al., 2007). In Michel et al. (2007), observers would gather an error between a visual target and visual feedback at the end of each trial in a reaching task, and would use the error for adequate control of reaching in the next trial. Effects of availability of an error between a feedback and target on temporal recalibration mechanisms should be examined systematically in future studies.

In summary, this study demonstrated that awareness of a temporal lag between a motor action and its visual feedback contributes strongly to motor–visual temporal recalibration. Consistent exposure to the temporal lag with its awareness induced substantial recalibration of motor–visual temporal order perception compared to that without its awareness. These results suggest that appropriate recalibration was achieved by high-level perceptual decision bias that results from registration of a temporal relationship with awareness. These findings provide new insight into the difference of mechanisms from multisensory temporal recalibration or prism adaptation.

### Conflict of interest statement

The authors declare that the research was conducted in the absence of any commercial or financial relationships that could be construed as a potential conflict of interest.
